# Integrating models of human behaviour between the individual and population levels to inform conservation interventions

**DOI:** 10.1098/rstb.2018.0053

**Published:** 2019-07-29

**Authors:** Andrew D. M. Dobson, Emiel de Lange, Aidan Keane, Harriet Ibbett, E. J. Milner-Gulland

**Affiliations:** 1 School of Geosciences, University of Edinburgh, Edinburgh EH8 9XP, UK; 2 Department of Zoology, University of Oxford, Oxford OX1 3SZ, UK

**Keywords:** social network analysis, agent-based model, conservation, information-sharing, predictive modelling, law enforcement

## Abstract

Conservation takes place within social–ecological systems, and many conservation interventions aim to influence human behaviour in order to push these systems towards sustainability. Predictive models of human behaviour are potentially powerful tools to support these interventions. This is particularly true if the models can link the attributes and behaviour of individuals with the dynamics of the social and environmental systems within which they operate. Here we explore this potential by showing how combining two modelling approaches (social network analysis, SNA, and agent-based modelling, ABM) could lead to more robust insights into a particular type of conservation intervention. We use our simple model, which simulates knowledge of ranger patrols through a hunting community and is based on empirical data from a Cambodian protected area, to highlight the complex, context-dependent nature of outcomes of information-sharing interventions, depending both on the configuration of the network and the attributes of the agents. We conclude by reflecting that both SNA and ABM, and many other modelling tools, are still too compartmentalized in application, either in ecology or social science, despite the strong methodological and conceptual parallels between their uses in different disciplines. Even a greater sharing of methods between disciplines is insufficient, however; given the impact of conservation on both the social and ecological aspects of systems (and vice versa), a fully integrated approach is needed, combining both the modelling approaches and the disciplinary insights of ecology and social science.

This article is part of the theme issue ‘Linking behaviour to dynamics of populations and communities: application of novel approaches in behavioural ecology to conservation’.

## 1. Introduction

Biodiversity loss and ecosystem degradation result from human actions such as over-harvesting of biological resources, destruction of habitat, and anthropogenic climate change [1,2]. Conservation is action taken to reduce the loss of biodiversity, to maintain the functioning of natural ecosystems, and to restore nature. While ecological knowledge is vital for successful conservation, the anthropogenic nature of these threats means that action to modify human behaviour and social systems is often necessary [3]. These systems operate across multiple scales, from global markets and governance institutions, to the behaviour of smallholder farmers [4]. Conservation interventions developed with little understanding of social system dynamics could result in simplistic and misguided approaches. More effective conservation, that seeks to influence the social drivers of ecological change, requires a more nuanced and predictive understanding of these drivers [5]. There have been recent calls for more predictive approaches in ecology, making ecological science more useful for real-world applications [6]. In conservation, there is a long history of using models to explore the effectiveness of different interventions (e.g. Population Viability Analysis [7,8] and the Incidence Function Model [9]). However, there is much less focus on the use of predictive modelling to understand human behavioural responses to conservation action [10].

Human behaviour is the object of inquiry in a vast number of academic disciplines with disparate epistemological perspectives and methodological approaches (e.g. [11]). Conservation scientists have predominantly drawn on economic and psychological models of individual behaviour [12]. For example, the Theory of Planned Behaviour from social psychology has been used extensively to understand individual behaviours and to design interventions [13]. Bounded Rationality, from economics, is also relevant, but has not been widely adopted in conservation [14]. Economic models of humans as rational actors have been used to understand hunting patterns [15]. Models from behavioural ecology are also applied to humans, analogous to the rational utility-maximizing models of economics but substituting fitness for utility. However, utility-maximizing frameworks need to be used with care, in the light of the important role of proximate mechanisms in determining the behaviour of both humans and other animals (e.g. psychological state; [16]).

Integrating the various bodies of knowledge about human behaviour with ecological data to produce more meaningful understanding of human-altered ecosystems and inform more effective conservation action, is an ongoing and challenging process [17]. One approach to integrating social and ecological knowledge that has been well developed is the modelling of social–ecological systems, which make explicit linkages between the ecological and social components of a system [18]. This enables predictions to be made about how changes in the social system might impact the ecosystem and vice versa [19]. However, the accuracy of these predictions depends on the degree to which knowledge of both the ecological and the social components is integrated into the model, and the manner in which this is achieved [20].

One technique which is very amenable to crossover between the ecological and social sciences is agent-based modelling (ABM), often referred to in ecology as individual-based modelling [21]. This sets rules for how individuals respond to their environment, allowing complex phenomena to emerge at the macro level. For example, ABMs have been used to model collective nest choice in ants [22], as well as racial segregation in urban neighbourhoods [23]. Social network analysis (SNA) is a complementary approach that provides theoretical frameworks for modelling the interactions between individuals, revealing how social structures influence individual behaviours and vice versa [24]. For example, SNA has been used to understand how innovations spread in populations of wild birds [25], and the spread of obesity in humans [26]. The two approaches differ principally in their perspective; an ABM is used to explore the outputs of complex systems (such as networks) by focusing on the behaviours of, and interactions between, individual components. In this view, the particular structures taken by the system are rarely the object of interest to the researcher. By contrast, in SNA—which is a suite of related techniques rather than a single method—the structural qualities of networks of individuals are explicitly regarded as determinants of group and/or individual behaviour and vice versa. Combining these two modelling approaches, such as by nesting ABMs within a social network, offers potentially rich insights into the behaviour of social groups. However, this has only rarely been attempted (though see [27] for an example from non-human epidemiology). One notable example from public health, which shares many characteristics with conservation (i.e. the goal of influencing the behaviour of groups of people), is the use of this integrated method to explore the effectiveness of anti-obesity interventions in social networks formed in schools [28].

One important basic mechanism influencing human behaviour is the flow of information. All theories of human behaviour recognize that people act on the basis of information received about the world [14], while acknowledging that the relationship between information receipt and subsequent behavioural change is not straightforward [29]. Not surprisingly, therefore, many conservation interventions aim to provide information in order to change individual behaviours. For example, law enforcement interventions may attempt to deter would-be rule-breakers by providing credible information about the risks and costs of punishment [30]. This information can come from direct experience or from communication with others. Many studies have shown that communication networks play a key role in determining who accesses certain information or adopts certain behaviours, and therefore that these networks determine socio-ecological outcomes (for a review in the context of natural resource management, see [31]). For example, in a Hawaiian fishery, a disconnection between two groups of fishers prohibited the spread of bycatch reduction techniques [32]. However, there is limited understanding within conservation science of the theory and practice of information flow, even though SNA is not an unfamiliar approach *per se*, and one which, ironically perhaps, has its origins in understanding the way that information moves through and influences human networks [33].

In this paper we explore how ABMs and SNAs, separately and in tandem, could be useful for understanding the dynamics of structured information flow. We examine the potential benefits of promoting a cross-over between the ecological and social sciences in conservation, where the emergent properties of individual actions inform system-level dynamics. We use a case study of the flow of information about the penalties for rule-breaking to illustrate how the two techniques may be used interactively to design an effective conservation intervention. We use insights from our work with communities in Cambodian protected areas to develop our analysis in the context of a real conservation situation [34]. We next reflect on the potential of SNAs and ABMs to improve our understanding of how individual decisions feed through into population-level dynamics, in the context of conservation science and practice. We close by reflecting on the future potential for cross-over between the ecological and social sciences in modelling human decision-making for conservation.

## 2. Social network analysis and agent-based modellings in ecology and social science

### (a) Agent-based modelling

An ABM is a bottom-up modelling approach in which the macro-scale characteristics of a complex system (for example, a biological community) are investigated by simulating the behaviour of constituent agents that follow pre-determined rules [35,36]. There may also be learning components, such that an agent's behaviour can be modified by experience [37,38].

Because small changes to starting conditions or behavioural rules could have profound downstream effects, ABMs are often better suited to exploring the potential mechanisms that are capable of generating observed phenomena and performing experiments *in silico* than to predicting real-world behaviour [39,40]. Furthermore, ABMs are context-specific simulations, and must therefore be designed for specific problems, meaning that generalizing their results can be challenging ([41], though see [42]). Nonetheless, ABMs have been very widely employed across the natural, social and economic sciences [41–43], in situations as diverse as the movement of people fleeing a building via a fire escape [44] and the potential response of the stock market to changes in trading policies [45]. In ecology, ABMs are often used to understand the dynamics of populations and communities of organisms, with agents representing either individuals (e.g. [46,47]) or other discrete units such as wolf packs [48]. Models combining humans and other animals can yield insights into human–wildlife conflicts (e.g. [49]) and the dynamics of harvesting (e.g. [50]), both of which have implications for conservation. ABMs for humans are conceptually identical to those for non-human animals, though in the case of humans there is much more scope for incorporating behaviour that has not been empirically observed, and hence to explore hypothetical scenarios.

### (b) Social network analysis

Humans, like other social animals, interact with others and form enduring relationships which influence how they behave. For example, through social interactions individuals come to learn new behaviours [51], but are also constrained by the need to conform to social norms [52]. In this way, many interacting individuals together make up societies which function in enduring and ordered ways. SNA is an analytical approach to studying social structures via the interactions between individuals, allowing these structures to be quantitatively described [24]. SNA techniques provide objective bases for testing hypotheses about relationships between network structures and emergent outcomes (e.g. the speed at which a problem can be solved by a network of people; [53]) and also allows researchers to explain how social structure influences, and is influenced by, the behaviours of individuals. In SNA, individuals are conceptualized as nodes connected by edges representing their interactions. These graphs are then analysed using network-theoretic concepts [24,54].

SNA has been used extensively in both behavioural ecology and the social sciences. In behavioural ecology, animal interactions are usually measured through observation, either directly or using proxies such as proximity at feeding sites [54]. In the social sciences, observational methods may also be used, but as human subjects are able to report on many of their own behaviours, survey-based methods are common [24]. Although theoretically the same analytical approaches may be used on social network data of any kind, in practice social scientists and behavioural ecologists tend to use different approaches. For example, to determine the influence of a social network on the spread of behaviours, behavioural ecologists commonly use Network-Based Diffusion Analysis [55]. While social scientists use similar approaches in studying network diffusion (e.g. [56]), they may also use Stochastic Actor-Oriented Models [57] or respondents' own perceptions of learning in their analyses (e.g. [58]). SNA is increasingly used in conservation, for example to understand how pro-conservation behaviours can be spread more effectively [31,59].

## 3. Case study—information flow to deter rule-breaking in hunting

### (a) Deterring rule-breaking in hunting

One of the most important drivers of defaunation, inside and outside protected areas (PAs), is the hunting of wild animals for food or sale, frequently described as ‘bushmeat hunting’ or ‘wild meat hunting’ [60–62]. Conservation law enforcement patrols are seen as a key line of defence against illegal hunting but, despite being a high funding priority, direct evidence of their effectiveness in deterring hunting within PAs is difficult to obtain ([63], though see [64]). Collecting first-hand information about the behaviour of hunters is often challenging or impossible because they may be unwilling to talk openly. The sensitivity of the topic means that even specialized questioning methods such as the randomized response technique and unmatched count technique [65] can fail to generate reliable data [66,67], and data from other sources, such as the records of ranger patrols, are difficult to interpret [63,68]. In this situation, modelling approaches based on behavioural ecology can provide an alternative means of exploring how patrols might be made more successful in preventing hunting, given specific assumptions about hunter behaviour and motivations.

Agent-based models have been applied to the related problem of sustainable hunting in both recreational and subsistence contexts. Ling & Milner-Gulland [69] investigated an Asiatic ibex (*Capra sibirica*) hunting system by coupling models of ibex ecology and the behaviour of human hunters, and found a complex set of dynamics in which the likelihood of sustainable equilibrium depended both on ibex behaviour (specifically the selection of relatively inaccessible locations) and the costs experienced by hunters. In a model of human settlement expansion in Amazonian Guyana, Iwamura *et al*. [70] simulated interactions between social and ecological systems driven by human nutritional requirements and resource availability in the environment. Feedback loops between animal and human population densities determined the equilibrium status of environmental quality and settlement size.

These models are complex and time-consuming to produce, but much simpler calculations based upon static indices of hunter effort (e.g. as a basic function of stock size) and population-level biological parameters for the prey are unlikely to adequately capture the inherent dynamics of hunting systems [71]. Indeed, hunting encompasses a nebulous set of activities that are typically motivated by the need to obtain food or income (either directly from the animals that are caught, or indirectly through the protection of crops), but also commonly shaped by institutions and social norms or customs [61]. Individual motivations and behaviour, which can vary significantly in response to environmental conditions, therefore contribute to, but are also shaped by, community norms and institutions—mediated by relationships between individuals. Planning an effective intervention at the community level therefore requires: (i) knowledge of existing social networks that mediate the transfer of information between individuals, and structure perceptions of norms; and (ii) the ability to model (forecast) the flow of new information with respect to a desired behavioural change.

**Figure 1.**
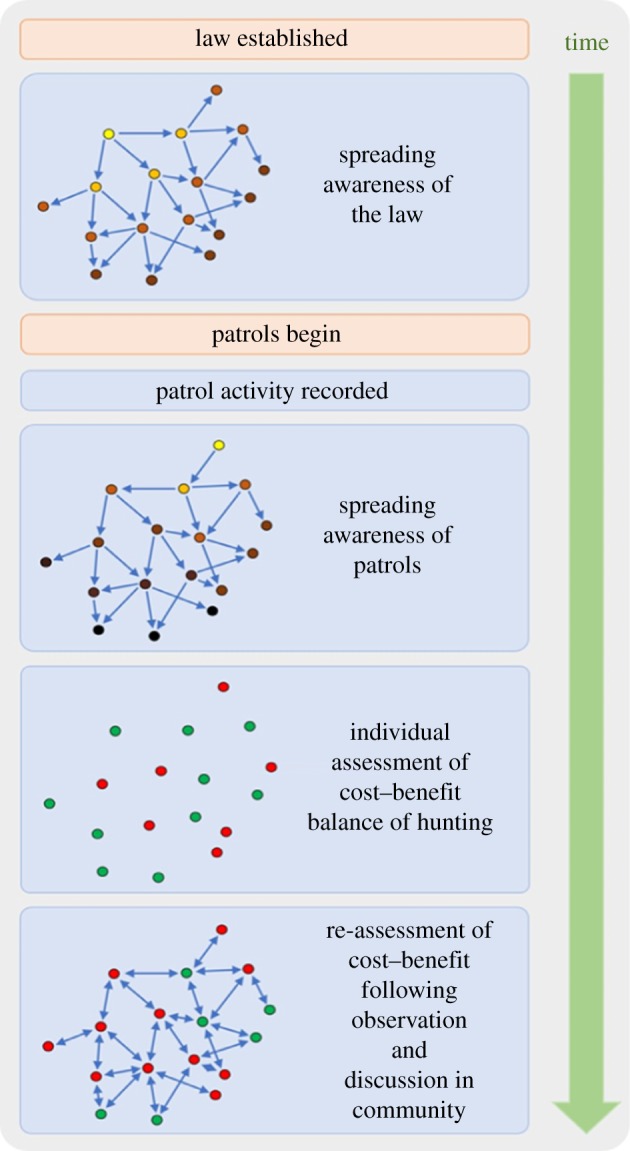
Flow diagram illustrating a potential series of behavioural processes that might occur following the institution of a statutory hunting prohibition, leading to the community-level response. Blue and beige shapes denote processes occurring within and outside the community, respectively. In the first two large boxes, the darkness of the point colour denotes the order in which the information is received (darker = later). In the other large boxes, green points are those that judge hunting to be cost-effective, and red points those that do not.

Many interventions that change behaviour do so by altering the positive and negative incentives that result from the interplay between economic and biological processes. In the case of hunting, economic and social gains from the acquisition of meat or other animal products are balanced against the direct and opportunity costs. However, while there have been a number of bio-economic models that attempt to quantify hunting behaviour in these terms (e.g. [72–74]), the role of deterrence following the imposition of a rule has had little attention in the conservation literature (e.g. [75]). This lack of research focus may derive in part from the complexity of the issue; as with any behavioural change, deterrence at the level of a community involves a series of both individual and social processes (figure 1), many of which are difficult to measure empirically. In these circumstances, combining SNA with an ABM offers a way to: (i) explore the emergent outcomes from sets of plausible starting conditions and behavioural rules, and (ii) identify the most influential individual processes, thereby setting priorities for targeted interventions.

### (b) Modelling the spread of information

To demonstrate how a combination of SNA and an ABM could facilitate the successful implementation of an intervention to deter rule-breaking at the level of a single community, we present a case study in which we simulate the spread of knowledge of the presence of law-enforcement patrols through communities with differing network structures. This process is only one among a series of steps that might characterize the full sequence from the creation of a rule to the outcome in terms of reduced rule-breaking at the community level (figure 1), but since our intention is to illustrate the modelling approach, we have kept the model simple. Nonetheless, its structure and parametrization are grounded in insights from our work with hunters in a Cambodian protected area [34]. We provide an overview of the model's construction here, with full details in the electronic supplementary material. With this approach we conserve the fundamental features of both SNA and an ABM, by modelling the flow of information through a connected network of individual agents. The elements of the model that build on SNA outputs relate to the properties of the network in terms of the presence and strength of the connections, while the agent-based element of the model means that individuals in the network differ in their propensity to spread and act on information (based on their probability of listening to and then passing on information received).

Non-spatial networks of 40 individuals were created in three types of community structure, which differed principally in the distribution of direct social contacts (‘degrees’ in SNA terminology) across individual members. Distributions were either highly, moderately or minimally skewed towards a theoretical extreme in which one individual is connected to everyone else, and no other pairs of individuals are connected. A variable proportion of individuals was then provided with knowledge of the presence of patrols. This initial knowledge of patrol presence was directly proportional to patrol effort, on the assumption that first-hand knowledge is gained by encountering patrols. We always selected the least-connected individuals, because in many tropical forest communities, most hunting is conducted by marginalized people living at forest edges (e.g. [76]). Where this is the case, knowledge of patrols may be concentrated in poorly connected individuals, though this scenario will not universally apply (we provide results in the electronic supplementary material for simulations where the best-connected individuals receive information first).

**Figure 2.**
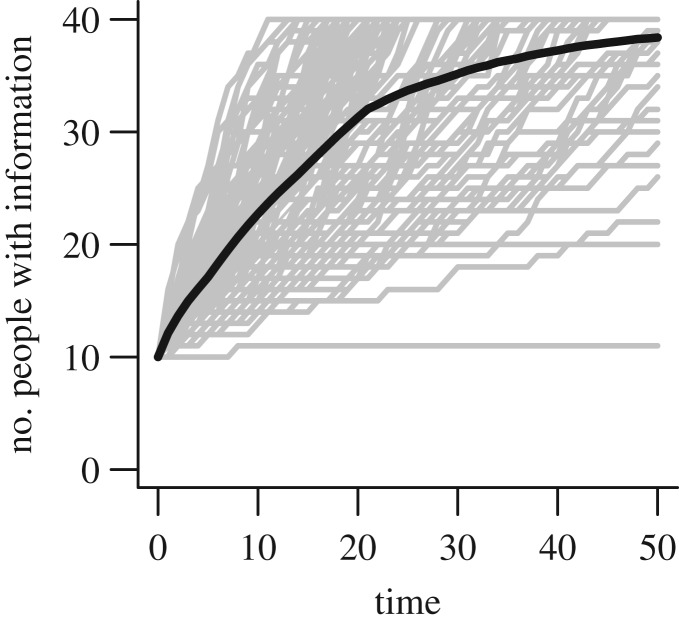
An example of information flow through a network of 40 individuals over time. The dark line represents the mean of 100 simulations, each of which is shown as a grey line. Parameter values as follows: *T* = 2, *E* = 0.25, *L* = 0.4.

We then simulated the flow of that knowledge through each network over fifty discrete time-steps of unspecified duration, under varying patrol effort, *E*, and rules for knowledge exchange. If a member of their immediate network had the information, the likelihood that an individual without it would receive it from them was controlled by two variables: (i) the probability of listening, *L* (and therefore the information-holder being able to pass on the information received), and (ii) the threshold number of knowledge-holding individuals to whom the recipient is directly connected, *T* (proxying the need for repeated independent transmission of information for it to be taken seriously). For each unique combination of variables, we repeated the simulations 100 times. We present the rate of information flow as the area under the curve (AUC) of the plot of cumulative receipt of information in the community over time (figure 2).

**Figure 3.**
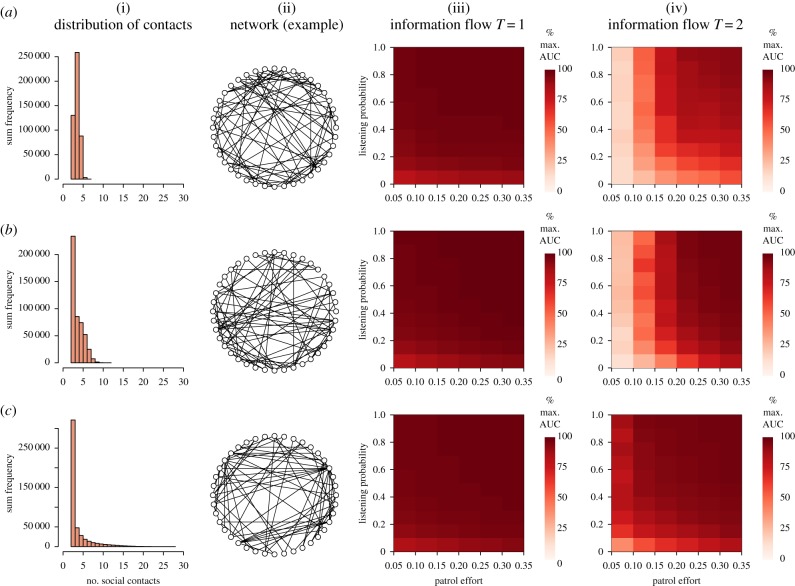
Impacts of network structure, listening probability (*L*), listening threshold (*T*) and patrol effort (*E*) on the rate of flow of information through networks of 40 individuals. The distribution of social contacts, which describes the evenness of connectedness among the community, is shown in column (i) and varies from lightly skewed (*a*), via moderately skewed (*b*) to highly skewed (*c*). When skewness is high, a small number of individuals are highly connected, while most individuals have only a small number of direct connections. Each histogram comprises data from 12 000 generated networks. Example network structures are shown in column (ii). Information flow is simulated through each of the networks for 100 replicates of each of the 120 combinations of *L*, *T* and *E*. Rate of information flow, characterized as the area under the curve (AUC) of plots of cumulative information accumulation over 50 time-steps (figure 2), is plotted against *L* and *E* in columns (iii) and (iv), for *T* = 1 and *T* = 2, respectively.

In general, information travelled fastest (highest AUC) when the distribution of social contacts was most skewed towards a handful of highly connected individuals, when the patrol effort (*E*) was high (meaning that more people started with the information), when the listening probability (*L*) was high, and when the listening threshold (*T*) was low. *E* and *L* both had significant positive effects on AUC (table 1; figure 3 columns (iii) and (iv)), but the effect of the interaction between these variables was dependent upon the listening threshold (*T*) and the distribution of social contacts. When *T* = 1, the interaction term was positively associated with AUC, but when *T* = 2 the association was significantly positive in networks with lightly skewed distributions, non-significant at moderate skews, and significantly negative with highly skewed distributions (table 1, far right column).

**Table 1.** Direction of the influence of listening probability (*L*) and patrol effort (*E*) on the rate of information flow through networks, from multiple linear regression analysis of AUC, for different values of listening threshold (*T*) and different distributions of social contacts. +, positive relationship; −, negative relationship (where +++/−−− represents *p* ≤ 0.01; +/− represents 0.01 < *p* ≤ 0.05, 0 represents *p* ≥ 0.05).

skew of social contacts distribution	*T* = 1			*T* = 2		
	*E*	*L*	E*L	*E*	*L*	E*L
low	+++	+++	−−−	+++	+	+++
moderate	+++	+++	−−−	+++	+++	0
high	+++	+++	−−−	+++	+++	−−−

These results imply that, when a recipient only needs to be connected to one information-holder to receive information, the increase in rate of information spread associated with increases in either patrol effort or propensity to listen is tempered when both increase at the same time. By contrast, when a potential recipient needs to be connected to two information-holders, and there is not much difference between individuals in their connectedness, increases in *E* and *L* reinforce each other. This phenomenon probably occurs due to the bounded nature of the response variable; AUC cannot exceed 2000 (the product of the number of time-steps and the number of people), so the functions linking AUC with *E* and *L* are saturating. When the distribution is highly skewed, AUC is close to 2000 even for the lowest values of *E* and *L*, such that there is far less potential for changes in either variable to effect a rise in AUC than when AUC starts at a lower value. These outputs encompass only a small proportion of the potential parameter space even for this simple model, however; different outcomes might have arisen had we altered the selection of individuals for information seeding, or increased the ranges of values of *T* and *E*.

## 4. Discussion and future perspectives

For a specific situation, the key strength of an ABM is its ability to follow the compound outcomes of a large number of interacting processes, in which the cause–effect relationships may be circular; the advantage of nesting agents in a social network within the ABM is that it constrains agent behaviour within a realistic social structure. Another useful consequence of incorporating network information into an ABM is the additional practice-relevant insights which this generates, such as with whom to seed information about the consequences of rule-breaking for maximum effectiveness, and what properties of an agent are most influential in determining their information-spreading effectiveness; such as their propensity to listen or their connectedness to others. We demonstrate these advantages with our ‘toy’ model. However, even for this very simple model simulating an isolated process, the outcomes were neither straightforward nor predictable.

Additional elements would be required in order to predict how a given information transfer process would translate into actual conservation outcomes, thereby making the model potentially useful for application in the real world. At the individual level, this could include more realistic variation between agents in their hunting behaviour, trust and trustworthiness (hence their likelihood of believing, and being believed, when passing on information) and risk profiles (hence their likelihood of acting upon the information), and their ability to switch to alternative hunting or non-hunting activities. There is also likely to be variation in their willingness to pass on information, and to whom. At the system level, prey population dynamics, the response of patrols to changes in hunter behaviour, and setting the scenario in a spatially explicit context, would all be important steps towards realism. Patrol responses would require a second set of agents in the ABM, as per Ling & Milner-Gulland [69,77], while prey dynamics could also be modelled as part of a spatially explicit ABM (cf. [50]). However, each of these additions is likely to dramatically increase the extent to which outcomes would be *a priori* unforeseeable, unless they constrain the system to the point at which insights are specific only to the individual system and its current circumstances.

There is an ongoing and well-recognized tension between complexity and simplicity in predictive modelling [39,78]. Heuristic insights tend to come from simple models which can capture the fundamental dynamics of a system [79]. On the other hand, detailed, spatially explicit, individual-based models can capture the emergent properties of a given social–ecological system that could lead to unintended consequences of conservation interventions [80]. As we recognize the importance of understanding social–ecological system dynamics in the context of a changing world (both socially and environmentally), the ability of ABMs to predict outside current conditions (so long as the basic properties of the agent remain constant) will become more useful, and potentially more worth the price of added complexity and specificity. This is in line with recent developments of ‘models of intermediate complexity’ which capture the main elements of complexity which are required for tactical decision-making in real-world situations [81].

We have highlighted two particular modelling approaches that are currently in individual use within ecology and social science, but which are rarely integrated (but see [28] for a public health example). We used the example of a particular conservation problem to show how their integration could bring benefits. But there is also a wider point: that methodological interchange between the social and ecological sciences is still more limited than it should be, even though they use the same modelling approaches in their own disciplines. For example, social and ecological feedbacks have been separately incorporated into systematic conservation planning, but not modelled together [82]; without incorporating both, the consequences of conservation action on the social–ecological system cannot be properly understood [5].

Part of the reason for this lack of integration is likely to be linguistic mismatches, such that it is not immediately clear that studies in ecology and social science are talking about the same thing (such as the terminology of individual-based versus agent-based model; [83]). Partly it may be that people still do not read papers in core disciplinary journals outside their own disciplines. To take one example, there is a growing literature on the determinants and consequences of network structure in ecology (e.g. in great tits, *Parus major*; [25,84]) and epidemiology (e.g. [27,85]) which is of direct relevance to conservationists' work on information-sharing between people, but as yet this linkage has not been explored. As conservation science becomes a discipline in its own right, one danger is that its interdisciplinary journals draw further away from its foundational disciplines, potentially leading to a lack of cross-fertilization. Interdisciplinarity is hard to achieve [86] and modelling is often viewed with suspicion by both conservationists and ecologists [87,88]. However, because conservationists operate within social–ecological systems, and aim to influence those systems in order to promote sustainability, it is important that these barriers to cross-disciplinary working are overcome.

The combined use of ABMs and SNAs to model the interactions between individual- and system-level dynamics, and predict the effect of conservation interventions on system dynamics, is one example of a way forward. This particular application will be further strengthened if we move beyond model-based prediction towards empirical testing of our hypothesized dynamics in the real world, within an adaptive management framework [89].

## Supplementary Material

Full methods and code for the model

## Data accessibility

This article has no additional data.
