# Supplementary material for: Integrating models of human behaviour between the individual and population levels to inform conservation interventions
Source: Philos Trans R Soc Lond B Biol Sci. 2019 Jul 29;374(1781):20180053. doi: 10.1098/rstb.2018.0053 (PMC6710576; doi:10.1098/rstb.2018.0053)
Supplement: Full methods and code for the model [file rstb20180053supp1.docx]

**Integrating models of human behaviour between the individual and population levels to inform conservation interventions**

Andrew Dobson, Emiel de Lange, Aidan Keane, Harriet Ibbett, E.J. Milner-Gulland

**Electronic Supplementary Material**

Contents

[1. FULL METHODS FOR THE MODEL 1](#_Toc3284927)

[2. IMPACT OF ALTERING THE ‘SEEDING’ RULE 5](#_Toc3284928)

[3. R CODE FOR THE MODEL 6](#_Toc3284929)

# 1. FULL METHODS FOR THE MODEL

We follow the ODD protocol (Overview, Design concepts, Details) of Grimm et al. (2006; 2010) to describe the model.

***Purpose***

The model is designed to demonstrate the impact of social network structure on the rate of information flow through a small human community.

***Entities, state variables and scales***

The model comprises a community of *N* people, connected in a Barabási-Albert network (Albert & Barabási 2002), in a non-spatial environment. Each member of the community is described by the state variable, *D*, the degree of connectedness, defined as the number of other people in the community network to whom they are socially connected. *D* does not change during any given simulation. At the start of each simulation, *S* people have the information that law enforcement patrols exist. This information is characterised as a binary state variable (i.e. individuals either know or do not know). Individuals may receive information only from others to whom they are directly connected.

***Process overview and scheduling***

At *t*=0, information is provided to *S* individuals (see *Inputs*). The model then proceeds in discrete time steps. At each step, individuals without the information have the potential to receive the information from those to which they are directly connected, with a probability, *P*, determined by two variables; the threshold number of information-holding individuals to whom they must be connected in order for information-sharing to take place, *T*, and their listening probability, *L*. Individuals receiving the information at time *t* cannot pass on the information until time *t*+1. Information is not forgotten once received.

***Design concepts***

*Interaction*: Individuals are assumed to have a single interaction with each of their direct connections every time step, and to have no chance of acquiring information from other individuals.

*Stochasticity*: Stochasticity is introduced at two stages; firstly during network creation, where the probability of connection to a given individual is drawn from one of three weighted distributions (see *Initialisation*), and secondly by probabilistic information transfer at each time step. The probability of receipt of information is mediated by *L* and *T* (see *Submodels*). We repeat simulations for each unique combination of *L* and *T* in order to provide ranges of outputs.

***Initialisation***

The network is constructed by adding the *N* people sequentially as network nodes, with each node initially joining to *m* existing nodes. During network creation, where 1<*n*≤*m*+1, there are *m* available nodes for the *n*th node to attach to. The network therefore does not grow until *n*>*m*+1. Beyond this point, the *n*th node has a choice of nodes to attach to (since the available nodes>*m*), and we vary the rule used to select these *m* nodes. We created three different sets of weights, *W*, each of which depends on the distribution of degrees (i.e. number of connections) across the existing members of the community. Where each added node is preferentially connected to the most highly connected nodes, the distribution is positively skewed. Where nodes connect to the most poorly connected nodes, the distribution becomes more uniform. The weights were then used to assign node connections in a probabilistic manner. Equations for the three sets of weights are as follows:

${W1}_{n}={(1-\frac{D_{n}}{\sum_{n=1}^{N} D_{n}})}^{200}$ Eq.1

${W2}_{n}={(1-\frac{D_{n}}{\sum_{n=1}^{N} D_{n}})}^{20}$ Eq.2

${W3}_{n}=\frac{D_{n}}{\sum_{n=1}^{N} D_{n}}$ Eq.3

Where *W*1*_n_*, *W*2*_n_* and *W*3*_n_* are the *n*th weights for each set. Degree distributions created from each set are shown in Figure 3, column (i) in the paper. *W*1 has the least skew; *W*3 the most.

***Input***

At *t*=0, *S* individuals are provided with the information that patrols exist. The value of *S* is determined by the following equation:

*S* = *EN*  Eq. 4

For 0<*E*<1

Where *E* is a measure of patrol effort.

The set *S* is chosen by sorting the members of the network in order of degree, and selecting the S least-connected individuals. Other methods could have been chosen; we use a single method for simplicity. Knowledge of patrols could be concentrated in poorly connected individuals in communities where most hunting is conducted by marginalised people living at forest edges (e.g. Harrison *et al*. 2015), though this scenario does not universally apply.

***Submodels***

*Information transfer*: At each time step, naïve individuals – those without the information – can receive the information if the number of information-bearing individuals to whom they are directly connected equals or exceeds *T*. If this condition is satisfied, the probability, *P*, that the naïve individual will receive the information in a given time step is provided by the following equation:

*P_n_* = *L*(1+*D_n_*-*T*) {if *P_n_* >1, *P_n_*=1} Eq. 5

**Simulations**

We ran the model for 50 time steps under different values of *L* (*n*=10), *T* (*n*=2) and *E* (*n*=6; Table ESM1), resulting in 120 combinations, and ran each combination using the three network structures. These simulations were repeated 100 times. We present the rate of information flow as the area under the curve (AUC) of the plot of cumulative receipt of information in the community over time (Figure xxx in the paper). When information flows quickly, AUC will approach 2000, the product of *N* and total *t*. Here we also present the plots as 3D surfaces, with bootstrapped 95% confidence intervals (Figure ESM1).

*Table ESM1. Parameters used in the case study.*

| **Parameter** | **Description** | **Symbol** | **Values** |
| --- | --- | --- | --- |
| Nodes | Number of individuals in the network | *N* | 40 |
| Links | Number of existing nodes to which each new node connects during network construction | *m* | 2 |
| Network structure | Algorithms used to construct the networks | *W* | (See Eqs 1-3) |
| Patrol effort | The relative presence of patrols - treated as the proportion of N who have knowledge of patrols at time *t*=0 | *E* | 0.1, 0.2, 0.3, 0.4, 0.5, 0.6 |
| Listening probability | Probability that an individual will 'absorb' information heard from directly connected individuals at each time step | *L* | 0.1, 0.2, 0.3, 0.4, 0.5, 0.6, 0.7, 0.8, 0.9, 1 |
| Listening threshold | Minimum number of information-bearing individuals to whom a node must be connected if they are to be receptive to information about patrols | *T* | 1, 2 |


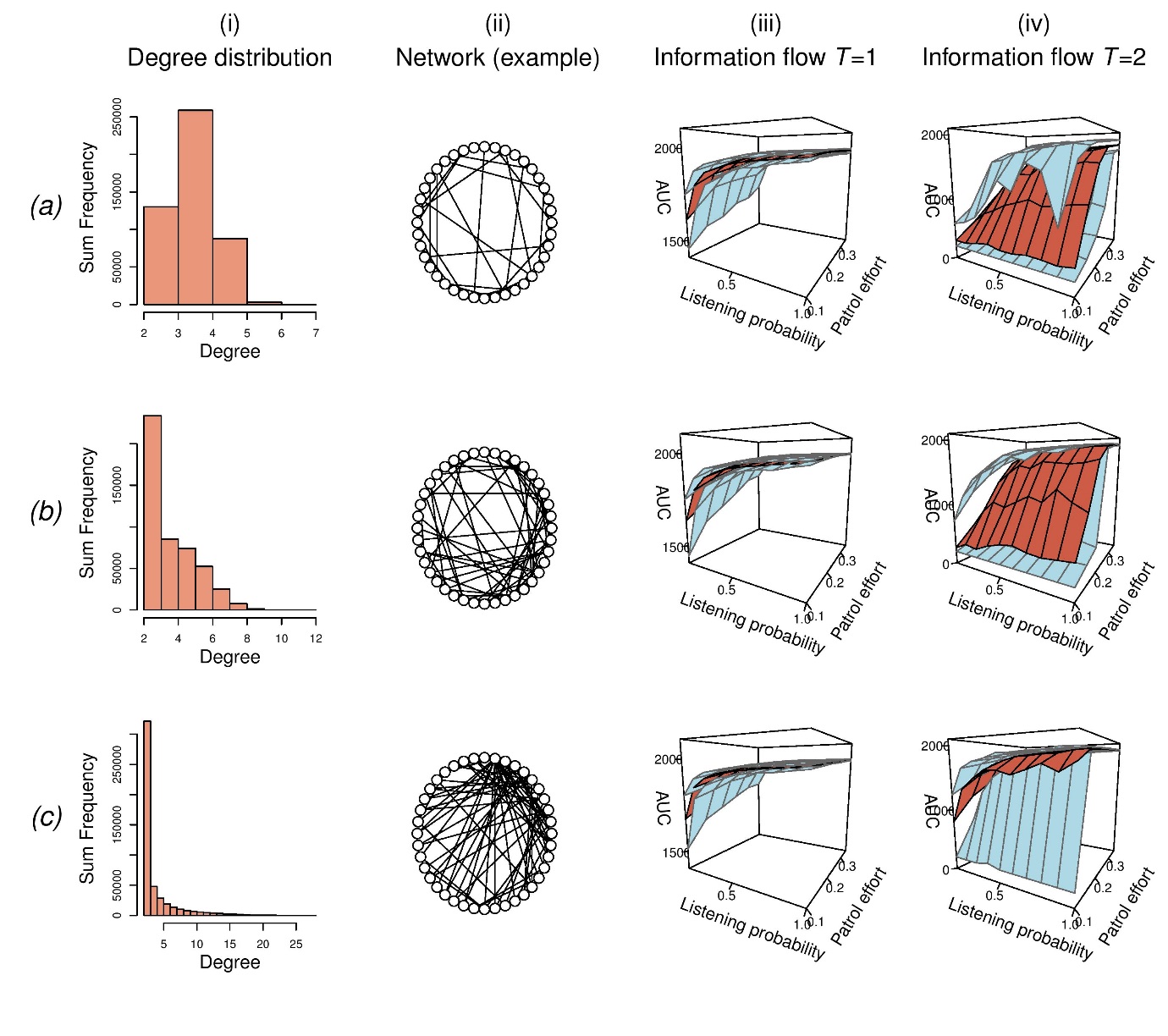


Figure ESM1. Equivalent of Figure 3, main paper, but with 3D surfaces to allow depiction of variation. Orange surfaces are means; grey surfaces are 95% confidence limits drawn from bootstrapped sample distributions (*n*=1000). Note differing z-axis limits in columns (iii) and (iv).

**References**

Albert, R., & Barabási, A. L. (2002). Statistical mechanics of complex networks. *Reviews of Modern Physics*, *74*(1), 47.

Grimm, V., Berger, U., Bastiansen, F., Eliassen, S., Ginot, V., Giske, J., Goss-Custard, J., Grand, T., Heinz, S., Huse, G., Huth, A., Jepsen, J.U., Jørgensen, C., Mooij, W.M., Müller, B., Pe’er, G., Piou, C., Railsback, S.F., Robbins, A.M., Robbins, M.M., Rossmanith, E., Rüger, N., Strand, E., Souissi, S., Stillman, R.A., Vabø, R., Visser, U., DeAngelis, D.L., 2006. A standard protocol for describing individual-based and agent-based models. *Ecological Modelling* 198, 115–126.

Grimm, V., Berger, U., DeAngelis, D. L., Polhill, J. G., Giske, J., & Railsback, S. F. (2010). The ODD protocol: a review and first update. *Ecological Modelling*, *221*(23), 2760-2768.

Harrison, M., Baker, J., Twinamatsiko, M., & Milner‐Gulland, E. J. (2015). Profiling unauthorized natural resource users for better targeting of conservation interventions. *Conservation Biology*, *29*(6), 1636-1646.

# 2. IMPACT OF ALTERING THE ‘SEEDING’ RULE

The following figure is the equivalent of Fig.3 in the main text, but instead of having the *x* least-connected individuals ‘seeded’ with the information of patrols, the *x* most-connected individuals are seeded. Information generally travels faster in these simulations (darker colours in columns iii and iv), as one would expect, though the overall pattern and the influences of the other parameters are unaffected.


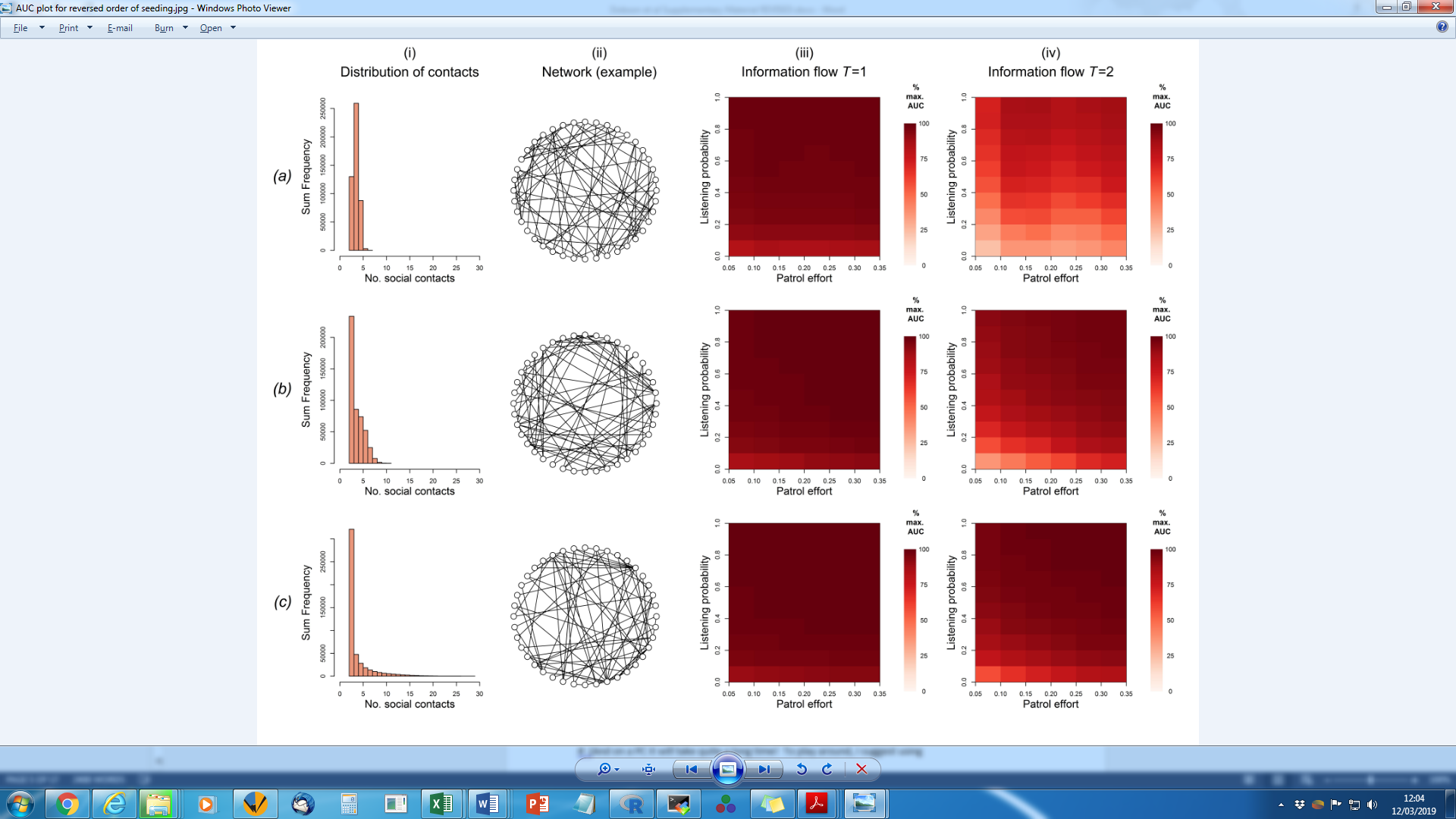


**Figure S1.** Impacts of network structure, listening probability (*L*), listening threshold (*T*) and patrol effort (*E*) on the rate of flow of information through networks of 40 individuals. The distribution of social contacts, which describes the evenness of connectedness among the community, is shown in column (i) and varies from lightly skewed (a), via moderately skewed (b) to highly skewed (c). When skewness is high, a small number of individuals are highly connected, whilst most individuals have only a small number of direct connections. Each histogram comprises data from 12000 generated networks. Example network structures are shown in column (ii). Information flow is simulated through each of the networks for 100 replicates of each of the 120 combinations of *L*, *T* and *E*. Rate of information flow, characterised as the area under the curve (AUC) of plots of cumulative information accumulation over 50 time-steps (see Figure 2), is plotted against *L* and *E* in columns (iii) and (iv), for *T*=1 and *T*=2, respectively.

# 3. R CODE FOR THE MODEL

# Code for Dobson et al. 2019, Phil. Trans.

# "Integrating models of human behaviour between the individual and population

# levels to inform conservation interventions"

# Users can alter 4 main parameters:

# 1. "nodes" - this is the number of individuals in the network

# 2. "m" - this is involved in the construction of the network, and provides

# the number of nodes to which each node is connected as it's added

# to the network

# 3. "ttr" - the number of discrete time-steps to run the simulation

# 4. "noreps" - the number of repetitions to be simulated under each unique

# combination of parameters.

# Other main parameters are varied within the existing code, but their values

# could obviously be changed if wanted. These are:

# (a) threshvals - threshold number of knowledge-holding individuals to whom

# the recipient of information must be directly connected

# before 'listening' occurs

# (b) patpresvals - patrol effort

# (c) abprobvals - likelihood of 'listening' (and subsequently potentially

# passing on information)

# The code will write datafiles and plots to the folder specified below.

# (And on a PC it will take quite a long time! To play around, I suggest using

# a small number of reps, i.e. <10)

setwd("what_folder")

nodes<-40

m<-2

ttr<-50

patpresvals<-c(0.1,0.15,0.2,0.25,0.3,0.35)

threshvals<-c(1,2)

abprobvals<-c(0.1,0.2,0.3,0.4,0.5,0.6,0.7,0.8,0.9,1)

randomseed<-F

pos<-F # If pos==T, the best-connected individuals receive info first

if(randomseed){

ran="RAN"

}else{

if(pos){

ran="NONRANpos"

}else{ran="NONRANneg"}

}

noreps<-100 # How many repetitions

dtable<-rep(0,(noreps+6))

t50rawtable<-rep(0,(noreps+6))

t50scaledtable<-rep(0,(noreps+6))

degreetable<-rep(0,4+nodes)

degreeset<-matrix(rep(0,(4+nodes)*noreps),nrow=noreps)

for (th in 1:length(threshvals)){

for (pa in 1:length(patpresvals)){

for (ab in 1:length(abprobvals)){

thresh<-threshvals[th]

patpres<-patpresvals[pa]

abprob<-abprobvals[ab]

seeds<-numeric(5)

alldegrees<-matrix(rep(0,nodes*noreps),nrow=nodes)

allinfo<-matrix(rep(0,(ttr+1)*noreps),nrow=noreps) # +1 because time starts at 0

for (k in 1:5){

for (rr in 1:noreps){

theset<-as.data.frame(matrix(rep(0,((nodes+3)*nodes)),nrow=nodes))

names(theset)[c(1,2,(nodes+3))]<-c("Node","Degree","Weight")

for (i in 1:nodes){

names(theset)[i+2]<-paste("N",i,"?",sep="")}

theset[,1]<-c(1:nodes)

theset$Degree[c(1:(m+1))]<-c(rep(m,(m+1)))

theset[1,4:(4+m-1)]<-1

for (q in 2:(m+1)){

theset[q,((c(1:(m+1))+2)[-q])]<-1

}

theset$Weight<-theset$Degree/sum(theset$Degree)

for (i in (m+2):nodes){ #~~~~~~~~~~~~~~~~~~~~~~~~~~~~~~~~~~~~~~ Start of i-loop

picks<-sample(c(1:(i-1)),m,replace=F,prob=theset$Weight[c(1:(i-1))])

theset$Degree[i]<-m

theset[i,picks+2]<-1

theset[picks,i+2]<-1

for (j in 1:(i-1)){

theset$Degree[j]<-sum(theset[j,c(3:(nodes+2))])}

if (k==1){

theset$Weight<-(1-(theset$Degree/sum(theset$Degree)))^200

theset$Weight<-theset$Weight/sum(theset$Weight)

}

if (k==2){

theset$Weight<-(1-(theset$Degree/sum(theset$Degree)))^50

theset$Weight<-theset$Weight/sum(theset$Weight)

}

if (k==3){

theset$Weight<-(1-(theset$Degree/sum(theset$Degree)))^20

theset$Weight<-theset$Weight/sum(theset$Weight)

}

if (k==4){

theset$Weight<-rep(1,length(theset[,1]))

theset$Weight<-theset$Weight/sum(theset$Weight)

}

if (k==5){

theset$Weight<-theset$Degree/sum(theset$Degree)

theset$Weight<-theset$Weight/sum(theset$Weight)

}} #~~~~~~~~~~~~~~~~~~~~~~~~~~~~~~~~~~~~~~~~~~~~~~~~~~~~~~~~~~~~~ End of i-loop

whichnode<-0

firstnodes<-as.data.frame(matrix(rep(0,2),nrow=1))

for (i in 1:nodes){

for (j in 1:sum(theset[i,c(3:(nodes+2))])){

whichnode<-which(theset[i,c(3:(nodes+2))]==1)[j]

firstnodes<-rbind(firstnodes,as.numeric(c(i,whichnode)))

}}

firstnodes<-firstnodes[-1,]

firstnodes$pair<-as.character(paste(firstnodes$V1,"-",firstnodes$V2,sep=""))

firstnodes$revpair<-as.character(paste(firstnodes$V2,"-",firstnodes$V1,sep=""))

firstnodes$V5<-rep(0,length(firstnodes[,1]))

for (i in 1:length(firstnodes[,1])){

if(firstnodes$V5[i]==0){

if(firstnodes$pair[i] %in% firstnodes$revpair){firstnodes$V5[which(firstnodes$revpair==firstnodes$pair[i])]<-1

}

}}

firstnodes<-firstnodes[-which(firstnodes$V5==1),]

firstnodes$deg1<-theset$Degree[firstnodes$V1]-1

firstnodes$deg2<-theset$Degree[firstnodes$V2]-1

X<-firstnodes$deg1

Y<-firstnodes$deg2

PCC<-(mean(X*Y)-(mean(X)*mean(Y)))/(sqrt(mean(X^2)-((mean(X))^2))*sqrt(mean(Y^2)-((mean(Y))^2)))

whoknows<-as.data.frame(matrix(rep(0,ttr*nodes),nrow=nodes))

nn<-rep(0,ttr)

for (i in 1:ttr){

nn[i]<-paste("t=",i,sep="")}

names(whoknows)<-nn

iseed<-rep(0,nodes)

if (randomseed){

iseed<-sample(c(0,1),nodes,prob=c((1-patpres),patpres),replace=T)

}else{

numbertoseed<-round(patpres*nodes)

if(pos){

toseed<-which(theset$Degree>=rev(sort(theset$Degree))[numbertoseed])

}else{

toseed<-which(theset$Degree<=(sort(theset$Degree))[numbertoseed])

}

if(length(toseed)>numbertoseed){toseed<-toseed[c(1:numbertoseed)]}

iseed[toseed]<-1

}

seeds[k]<-sum(iseed)

thelist=0

for (j in 1:ttr){

foundout<-rep(0,nodes)

nodeorder<-sample(1:nodes,nodes,replace=F)

for (i in nodeorder){

if(j==1){thelist<-iseed}else{thelist<-whoknows[,j-1]}

if(thelist[i]!=1){

palswhoknow<-which((thelist==1) & as.double(theset[i,3:(nodes+2)])==1)

numpals<-length(palswhoknow)

if (numpals>=thresh){

threshpals<-1+(numpals-thresh)

absorbprob=abprob*threshpals

if (absorbprob>1){

absorbprob<-1

}

foundout[i]<-sample(c(0,1),1,prob=c((1-absorbprob),absorbprob),replace=F)

}else{foundout[i]<-0

}

}else{foundout[i]<-1

}

}

whoknows[,j]<-foundout

}

for (i in 1:nodes){

if (whoknows[i,1]<iseed[i]){print("Someone's forgotten something")}

}

for (j in 2:ttr){

for (i in 1:nodes){

if (whoknows[i,j]<whoknows[i,j-1]){print("Someone's forgotten something")}

}}

alldegrees[,rr]<-theset$Degree

allinfo[rr,]<-colSums(cbind(iseed,whoknows))

thecount<-((th-1)*length(patpresvals)*length(abprobvals)*5*noreps)+

((pa-1)*length(abprobvals)*5*noreps)+

((ab-1)*5*noreps)+

((k-1)*noreps)+rr

print(paste("Rep number ",thecount," of ",length(threshvals)*length(patpresvals)*length(abprobvals)*5*noreps,sep=""))

} #~~~~~~~~~~~~~~~~~~~~~~~~~~~~~~~~~~~~~~~~~~~~~~~~~~~~~~~~~~~~~~ End of rr loop

edge<-2

span<-5

pointset<-as.data.frame(matrix(numeric(3*nodes),nrow=nodes))

names(pointset)<-c("Node","X","Y")

pointset$Node<-c(1:nodes)

for (i in 1:nodes){

pointset$X[i]<-edge+span+(span*sin(((360*i)/nodes)*(pi/180)))

pointset$Y[i]<-edge+span+(span*cos(((360*i)/nodes)*(pi/180)))

}

averagedegrees<-rep(0,nodes)

for (dd in 1:nodes){averagedegrees[dd]<-mean(alldegrees[dd,])}

averageinfo<-rep(0,(ttr+1))

for (dd in 1:(ttr+1)){averageinfo[dd]<-mean(allinfo[,dd])}

integ<-rowSums(allinfo)

t50raw<-numeric(noreps)

t50scaled<-numeric(noreps)

naive<-nodes-seeds[k]

for (tt in 1:noreps){

t50raw[tt]<-which(allinfo[tt,]>=(nodes/2))[1]

t50scaled[tt]<-which(allinfo[tt,]>=(seeds[k]+(0.5*naive)))[1]

}

if(k==1 & th==1 & pa==1 & ab==1){

dtable[1]<-thresh->degreetable[1]

dtable[2]<-patpres->degreetable[2]

dtable[3]<-abprob->degreetable[3]

dtable[4]<-1->degreetable[4]

t50rawtable[1]<-dtable[1]

t50rawtable[2]<-dtable[2]

t50rawtable[3]<-dtable[3]

t50rawtable[4]<-dtable[4]

t50scaledtable[1]<-dtable[1]

t50scaledtable[2]<-dtable[2]

t50scaledtable[3]<-dtable[3]

t50scaledtable[4]<-dtable[4]

dtable[5:(noreps+4)]<-integ

degreetable[5:(nodes+4)]<-averagedegrees

t50rawtable[5:(noreps+4)]<-t50raw

t50scaledtable[5:(noreps+4)]<-t50scaled

degreeset[,1]<-thresh

degreeset[,2]<-patpres

degreeset[,3]<-abprob

degreeset[,4]<-1

degreeset[,5:(nodes+4)]<-t(alldegrees)

}else{

dtable<-rbind(dtable,c(thresh,patpres,abprob,k,integ,rep(0,2)))

t50rawtable<-rbind(t50rawtable,c(thresh,patpres,abprob,k,t50raw,rep(0,2)))

t50scaledtable<-rbind(t50scaledtable,c(thresh,patpres,abprob,k,t50scaled,rep(0,2)))

degreetable<-rbind(degreetable,c(thresh,patpres,abprob,k,averagedegrees))

degreeset<-rbind(degreeset,

cbind(rep(thresh,noreps),rep(patpres,noreps),rep(abprob,noreps),rep(k,noreps),t(alldegrees)))

}

} #~~~~~~~~~~~~~~~~~~~~~~~~~~~~~~~~~~~~~~~~~~~~~~~~~~~~~~~~~~~~~~ End of k loop

} #~~~~~~~~~~~~~~~~~~~~~~~~~~~~~~~~~~~~~~~~~~~~~~~~~~~~~~~~~~~~~ End of ab loop

} #~~~~~~~~~~~~~~~~~~~~~~~~~~~~~~~~~~~~~~~~~~~~~~~~~~~~~~~~~~~~~ End of pa loop

} #~~~~~~~~~~~~~~~~~~~~~~~~~~~~~~~~~~~~~~~~~~~~~~~~~~~~~~~~~~~~~ End of th loop

for(jj in 1:dim(dtable)[1]){

dtable[jj,(noreps+5)]<-mean(dtable[jj,5:(noreps+4)])

dtable[jj,(noreps+6)]<-sd(dtable[jj,5:(noreps+4)])

t50rawtable[jj,(noreps+5)]<-mean(t50rawtable[jj,5:(noreps+4)])

t50rawtable[jj,(noreps+6)]<-sd(t50rawtable[jj,5:(noreps+4)])

t50scaledtable[jj,(noreps+5)]<-mean(t50scaledtable[jj,5:(noreps+4)])

t50scaledtable[jj,(noreps+6)]<-sd(t50scaledtable[jj,5:(noreps+4)])

}

write.csv(dtable,"dtable50.csv")

write.csv(degreetable,"degreetable50.csv")

write.csv(degreeset,"degreeset50.csv")

################################################################################

################################################################################

################################################################################

################################################################################

#~~~~~~~~~~~~~~~~~~~~~~~~~~~~~~~~~~~~~~~~~~~~~~~~~~~~~~~~~~~~~~~~~~~~~~~~~~~~~~#

#~~~~~~~~~~~~~~~~~~~~~~~~~~~~~~~~~~~~~~~~~~~~~~~~~~~~~~~~~~~~~~~~~~~~~~~~~~~~~~#

#~~~~~~~~~~~~~~~~~~~~~~~~~~~~~~~~~~~~~~~~~~~~~~~~~~~~~~~~~~~~~~~~~~~~~~~~~~~~~~#

#~~~~~~~~~~~~~~~~~~~~~~~~~~~~~~~~ PLOTTING ~~~~~~~~~~~~~~~~~~~~~~~~~~~~~~~~~~~~#

#~~~~~~~~~~~~~~~~~~~~~~~~~~~~~~~~~~~~~~~~~~~~~~~~~~~~~~~~~~~~~~~~~~~~~~~~~~~~~~#

#~~~~~~~~~~~~~~~~~~~~~~~~~~~~~~~~~~~~~~~~~~~~~~~~~~~~~~~~~~~~~~~~~~~~~~~~~~~~~~#

#~~~~~~~~~~~~~~~~~~~~~~~~~~~~~~~~~~~~~~~~~~~~~~~~~~~~~~~~~~~~~~~~~~~~~~~~~~~~~~#

################################################################################

################################################################################

################################################################################

################################################################################

library(RColorBrewer)

#dtable<-read.csv("dtable50.csv")[,-1]

#degreetable<-read.csv("degreetable50.csv")[,-1]

#degreeset<-read.csv("degreeset50.csv")[,-1]

dtable$V105<-0

dtable$V106<-0

#Adjust dtable to remove the number of individuals to whom the info was seeded -

# otherwise this gives you potential max AUC greater than 2000 (i.e. >100%)

seedstotake<-numeric(length(dtable[,1]))

for (i in 1:length(dtable[,1])){

seedstotake[i]<-round(dtable[i,2]*nodes)

}

for (i in 1:length(dtable[,1])){

for (j in 5:noreps+4){

dtable[i,j]<-dtable[i,j]-seedstotake[i]

}}

for (i in 1:dim(dtable)[1]){

dtable$V105[i]<-mean(as.double(dtable[i,c(5:(noreps+4))]))

dtable$V106[i]<-sd(as.double(dtable[i,c(5:(noreps+4))]))

}

#~~~~~~~~~~~~~~~~~~~~~~~ Now plot everything in 2D ~~~~~~~~~~~~~~~~~~~~~~~~~~~~#

bootsize<-1000 # for use with bootstrap

# get the zlims

zmin<-c(1400,0)#1min(dtable[,c(5:(noreps+4))])

zmax<-2100#max(dtable[,c(5:(noreps+4))])

# Layout options

layoutA<-matrix(c(1,1,1,1,2,2,2,2,3,3,3,3,4,4,6,6,6,6,7,7,

1,1,1,1,2,2,2,2,3,3,3,3,4,4,6,6,6,6,7,7,

1,1,1,1,2,2,2,2,3,3,3,3,5,5,6,6,6,6,8,8,

1,1,1,1,2,2,2,2,3,3,3,3,5,5,6,6,6,6,8,8,

9,9,9,9,10,10,10,10,11,11,11,11,12,12,14,14,14,14,15,15,

9,9,9,9,10,10,10,10,11,11,11,11,12,12,14,14,14,14,15,15,

9,9,9,9,10,10,10,10,11,11,11,11,13,13,14,14,14,14,16,16,

9,9,9,9,10,10,10,10,11,11,11,11,13,13,14,14,14,14,16,16,

17,17,17,17,18,18,18,18,19,19,19,19,20,20,22,22,22,22,23,23,

17,17,17,17,18,18,18,18,19,19,19,19,20,20,22,22,22,22,23,23,

17,17,17,17,18,18,18,18,19,19,19,19,21,21,22,22,22,22,24,24,

17,17,17,17,18,18,18,18,19,19,19,19,21,21,22,22,22,22,24,24),nrow=12,byrow=T)

layoutB<-matrix(c(1,2,3,4,

5,6,7,8,

9,10,11,12),nrow=3,byrow=T)

layoutB<-matrix(c(1,1,1,1,2,2,2,2,3,3,3,3,4,5,5,5,5,6,

1,1,1,1,2,2,2,2,3,3,3,3,4,5,5,5,5,6,

1,1,1,1,2,2,2,2,3,3,3,3,4,5,5,5,5,6,

1,1,1,1,2,2,2,2,3,3,3,3,4,5,5,5,5,6,

7,7,7,7,8,8,8,8,9,9,9,9,10,11,11,11,11,12,

7,7,7,7,8,8,8,8,9,9,9,9,10,11,11,11,11,12,

7,7,7,7,8,8,8,8,9,9,9,9,10,11,11,11,11,12,

7,7,7,7,8,8,8,8,9,9,9,9,10,11,11,11,11,12,

13,13,13,13,14,14,14,14,15,15,15,15,16,17,17,17,17,18,

13,13,13,13,14,14,14,14,15,15,15,15,16,17,17,17,17,18,

13,13,13,13,14,14,14,14,15,15,15,15,16,17,17,17,17,18,

13,13,13,13,14,14,14,14,15,15,15,15,16,17,17,17,17,18)

,nrow=12,byrow=T)

layoutC<-matrix(c(1,1,3,3,3,3,4,4,6,6,6,6,7,7,

1,1,3,3,3,3,4,4,6,6,6,6,7,7,

2,2,3,3,3,3,5,5,6,6,6,6,8,8,

2,2,3,3,3,3,5,5,6,6,6,6,8,8,

9,9,11,11,11,11,12,12,14,14,14,14,15,15,

9,9,11,11,11,11,12,12,14,14,14,14,15,15,

10,10,11,11,11,11,13,13,14,14,14,14,16,16,

10,10,11,11,11,11,13,13,14,14,14,14,16,16,

17,17,19,19,19,19,20,20,22,22,22,22,23,23,

17,17,19,19,19,19,20,20,22,22,22,22,23,23,

18,18,19,19,19,19,21,21,22,22,22,22,24,24,

18,18,19,19,19,19,21,21,22,22,22,22,24,24)

,byrow=T,nrow=12)

layoutD<-matrix(c(1,1,3,3,3,3,3,3,4,4,4,6,6,6,6,6,6,7,7,7,

1,1,3,3,3,3,3,3,4,4,4,6,6,6,6,6,6,7,7,7,

25,25,3,3,3,3,3,3,4,4,4,6,6,6,6,6,6,7,7,7,

2,2,3,3,3,3,3,3,5,5,5,6,6,6,6,6,6,8,8,8,

2,2,3,3,3,3,3,3,5,5,5,6,6,6,6,6,6,8,8,8,

25,25,3,3,3,3,3,3,5,5,5,6,6,6,6,6,6,8,8,8,

9,9,11,11,11,11,11,11,12,12,12,14,14,14,14,14,14,15,15,15,

9,9,11,11,11,11,11,11,12,12,12,14,14,14,14,14,14,15,15,15,

25,25,11,11,11,11,11,11,12,12,12,14,14,14,14,14,14,15,15,15,

10,10,11,11,11,11,11,11,13,13,13,14,14,14,14,14,14,16,16,16,

10,10,11,11,11,11,11,11,13,13,13,14,14,14,14,14,14,16,16,16,

25,25,11,11,11,11,11,11,13,13,13,14,14,14,14,14,14,16,16,16,

17,17,19,19,19,19,19,19,20,20,20,22,22,22,22,22,22,23,23,23,

17,17,19,19,19,19,19,19,20,20,20,22,22,22,22,22,22,23,23,23,

25,25,19,19,19,19,19,19,20,20,20,22,22,22,22,22,22,23,23,23,

18,18,19,19,19,19,19,19,21,21,21,22,22,22,22,22,22,24,24,24,

18,18,19,19,19,19,19,19,21,21,21,22,22,22,22,22,22,24,24,24,

25,25,19,19,19,19,19,19,21,21,21,22,22,22,22,22,22,24,24,24)

,byrow=T,nrow=18)

layoutletter<-"B"

whichlayout<-get(paste("layout",layoutletter,sep=""))

#pdf(paste("2D-AUC_",ceiling(proc.time()[3]),"_layout",layoutletter,".pdf",sep=""),height=12,width=16)

jpeg(paste("NH_2D-AUC_",ceiling(proc.time()[3]),"_layout",layoutletter,".jpg",sep=""),height=12*700,width=16*700,res=700)

par(mfrow=c(3,4),oma=c(3.25,6,5.5,1),mar=c(5,4,2,2))

layout(whichlayout)

#

axsize<-1.3

axlabsize<-1.1

for (k in c(1,3,5)){

if (identical(whichlayout,layoutB)==F){par(mar=c(0,2,0,1))}

degs<-degreeset[which(degreeset[,4]==k),c(5:(nodes+4))]

# plot distribution here

par(mar=c(5,4,2,2))

#hist(unlist(degs),length(unique(unlist(degs))),main="",xlab="",ylab="",bty='o',col="darksalmon",cex=axsize)

hist(unlist(degs),length(unique(unlist(degs))),main="",xlab="",ylab="",bty='o',xlim=c(0,30),col="darksalmon",cex=axsize)

# degs<-degreetable[which(degreetable[,4]==k),c(5:(nodes+4))]

# avedeg<-colSums(degs)/length(degs[,1])

# # plot distribution here

# hist(avedeg,length(unique(avedeg)),main="",xlab="",ylab="",bty='o',col="darksalmon")

mtext("No. social contacts",1,2.5,cex=axlabsize)

mtext("Sum Frequency",2,3,cex=axlabsize)

letterdist<-5.5

numberheight<-5

lettsize<-1.6

numbersize<-1.4

headersize<-1.4

headerheight<-2.5

if (k==1){

mtext("Distribution of contacts",3,headerheight,cex=headersize)

mtext("(i)",3,numberheight,cex=numbersize)

}

if (k==1){

mtext(expression(italic("(a)")),2,letterdist,las=2,cex=lettsize)

}

if (k==2){

mtext(expression(italic("(b)")),2,letterdist,las=2,cex=lettsize)

}

if (k==3){

mtext(expression(italic("(b)")),2,letterdist,las=2,cex=lettsize)

}

if (k==4){

mtext(expression(italic("(d)")),2,letterdist,las=2,cex=lettsize)

}

if (k==5){

mtext(expression(italic("(c)")),2,letterdist,las=2,cex=lettsize)

}

#~~~~~~~~~~~~~~~~~~~~~~~~~~~~~~~~~~~~~~~~~~~~~~~~~~~~~~~~~~~~~~~~~~~~~~~~~~~~~~#

#~~~~~~~~~~~~~~~~~~~~~~~~~~~~~~~~~~~~~~~~~~~~~~~~~~~~~~~~~~~~~~~~~~~~~~~~~~~~~~#

#~~~~~~~~~~~~~~~~~~~~~~~~~~~~~~~~~~~~~~~~~~~~~~~~~~~~~~~~~~~~~~~~~~~~~~~~~~~~~~#

#~~~~~~~~~~~~~~~~~~~~~~~~~~~~~~~~~~~~~~~~~~~~~~~~~~~~~~~~~~~~~~~~~~~~~~~~~~~~~~#

#~~~~~~~~~~~~~~~~~~~~~~~~~~~~~~~~~~~~~~~~~~~~~~~~~~~~~~~~~~~~~~~~~~~~~~~~~~~~~~#

#~~~~~~~~~~~~~~~~~~~~~~~~~~~~~~~~~~~~~~~~~~~~~~~~~~~~~~~~~~~~~~~~~~~~~~~~~~~~~~#

#~~~~~~~~~~~~~~~~~~~~~~~~~~~~~~~~~~~~~~~~~~~~~~~~~~~~~~~~~~~~~~~~~~~~~~~~~~~~~~#

#~~~~~~~~~~~~~~~~~~~~~~~~~~~~~~~~~~~~~~~~~~~~~~~~~~~~~~~~~~~~~~~~~~~~~~~~~~~~~~#

#~~~~~~~~~~~~~~~~~~~~~~~~~ Network stuff and plots ~~~~~~~~~~~~~~~~~~~~~~~~~~~~#

theset<-as.data.frame(matrix(rep(0,((nodes+3)*nodes)),nrow=nodes))

names(theset)[c(1,2,(nodes+3))]<-c("Node","Degree","Weight")

for (i in 1:nodes){

names(theset)[i+2]<-paste("N",i,"?",sep="")}

theset[,1]<-c(1:nodes)

theset$Degree[c(1:(m+1))]<-c(rep(m,(m+1)))

theset[1,4:(4+m-1)]<-1

for (q in 2:(m+1)){

theset[q,((c(1:(m+1))+2)[-q])]<-1

}

theset$Weight<-theset$Degree/sum(theset$Degree)

# Subsequent nodes

for (i in (m+2):nodes){ #~~~~~~~~~~~~~~~~~~~~~~~~~~~~~~~~~~~~~~~~~ Start of i-loop

picks<-sample(c(1:(i-1)),m,replace=F,prob=theset$Weight[c(1:(i-1))])

theset$Degree[i]<-m

theset[i,picks+2]<-1 # connection nodes for the ith node

theset[picks,i+2]<-1 # connection nodes for the nodes that node i has just connected to

# Recalculate degree and weight for everything

for (j in 1:(i-1)){

theset$Degree[j]<-sum(theset[j,c(3:(nodes+2))])}

#~~ degrees here...

if (k==1){

theset$Weight<-(1-(theset$Degree/sum(theset$Degree)))^200

theset$Weight<-theset$Weight/sum(theset$Weight)

}

if (k==2){

theset$Weight<-(1-(theset$Degree/sum(theset$Degree)))^50

theset$Weight<-theset$Weight/sum(theset$Weight)

}

if (k==3){

theset$Weight<-(1-(theset$Degree/sum(theset$Degree)))^20

theset$Weight<-theset$Weight/sum(theset$Weight)

}

if (k==4){

theset$Weight<-rep(1,length(theset[,1]))

theset$Weight<-theset$Weight/sum(theset$Weight)

}

if (k==5){

theset$Weight<-theset$Degree/sum(theset$Degree)

theset$Weight<-theset$Weight/sum(theset$Weight)

}} #~~~~~~~~~~~~~~~~~~~~~~~~~~~~~~~~~~~~~~~~~~~~~~~~~~~~~~~~~~~~~~~ End of i-loop

#~~~~~~~~~~~~~~ The network ~~~~~~~~~~~~~~~#

edge<-2

span<-5

pointset<-as.data.frame(matrix(numeric(3*nodes),nrow=nodes))

names(pointset)<-c("Node","X","Y")

pointset$Node<-c(1:nodes)

nodesran<-sample(c(1:nodes),nodes,replace=F)

par(mar=c(3,0,0,0))

for (i in 1:nodes){

pointset$X[nodesran[i]]<-edge+span+(span*sin(((360*i)/nodes)*(pi/180)))

pointset$Y[nodesran[i]]<-edge+span+(span*cos(((360*i)/nodes)*(pi/180)))

}

plot(0,

ylim=c(min(pointset$Y)*0.8,max(pointset$Y)*1.2),

xlim=c(min(pointset$X)*0.8,max(pointset$X)*1.2),

yaxt='n',xaxt='n',ylab="",xlab="",bty='n')

# Add links

for (i in 1:nodes){

for (j in 1:sum(theset[i,c(3:(nodes+2))])){

xx<-c(pointset$X[i],pointset$X[(which(theset[i,c(3:(nodes+2))]==1))[j]])

yy<-c(pointset$Y[i],pointset$Y[(which(theset[i,c(3:(nodes+2))]==1))[j]])

lines(yy~xx)

}}

points(pointset$Y~pointset$X,yaxt='n',xaxt='n',ylab="",xlab="",bty='n',pch=21,bg="white",cex=2)

if(k==1){

mtext("Network (example)",3,headerheight-2,cex=headersize)

mtext("(ii)",3,numberheight-2,cex=numbersize)

}

#~~~~~~~~~~~~~~~~~~~~~~~~~~~~~~~~~~~~~~~~~~~~~~~~~~~~~~~~~~~~~~~~~~~~~~~~~~~~~~#

#~~~~~~~~~~~~~~~~~~~~~~~~~~~~~~~~~~~~~~~~~~~~~~~~~~~~~~~~~~~~~~~~~~~~~~~~~~~~~~#

#~~~~~~~~~~~~~~~~~~~~~~~~~~~~~~~~~~~~~~~~~~~~~~~~~~~~~~~~~~~~~~~~~~~~~~~~~~~~~~#

#~~~~~~~~~~~~~~~~~~~~~~~~~~~~~~~~~~~~~~~~~~~~~~~~~~~~~~~~~~~~~~~~~~~~~~~~~~~~~~#

#~~~~~~~~~~~~~~~~~~~~~~~~~~~~~~~~~~~~~~~~~~~~~~~~~~~~~~~~~~~~~~~~~~~~~~~~~~~~~~#

#~~~~~~~~~~~~~~~~~~~~~~~~~~~~~~~~~~~~~~~~~~~~~~~~~~~~~~~~~~~~~~~~~~~~~~~~~~~~~~#

#~~~~~~~~~~~~~~~~~~~~~~~~~~~~~~~~~~~~~~~~~~~~~~~~~~~~~~~~~~~~~~~~~~~~~~~~~~~~~~#

# Now plot the AUC views, one for each th value

for (th in 1:2){

par(mar=c(5,4,2,2))

indat<-as.data.frame(dtable[which(dtable[,4]==k & dtable[,1]==threshvals[th]),])

names(indat)[1:4]<-c("th","pa","ab","k")

auc<-indat[,(noreps+5)]

aucSD<-rep(0,length(auc))

for (gg in 1:length(auc)){

aucSD[gg]<-sd(indat[gg,c(5:(noreps+4))])

}

# Need to transform the dataset to fit with the persp function requirements

pavals<-sort(unique(indat$pa))

abvals<-sort(unique(indat$ab))

AUC<-matrix(rep(0,length(pavals)*length(abvals)),nrow=length(abvals))

for (i in 1:length(abvals)){

for (j in 1:length(pavals)){

AUC[i,j]<-auc[which(indat$ab==abvals[i] & indat$pa==pavals[j])]

}}

# Now generate data for errors

# bootstrapped 95%

AUC95upper<-matrix(rep(0,length(pavals)*length(abvals)),nrow=length(abvals))

AUC95lower<-matrix(rep(0,length(pavals)*length(abvals)),nrow=length(abvals))

for (i in 1:length(abvals)){

for (j in 1:length(pavals)){

dats<-indat[which(indat$ab==abvals[i] & indat$pa==pavals[j]),c(5:(noreps+4))]

booted<-sample(dats,bootsize,replace=T)

AUC95lower[i,j]<-as.double(sort(booted)[round(bootsize*0.025)])

AUC95upper[i,j]<-as.double(sort(booted)[round(bootsize*0.975)])

}}

AUClowP<-100-((AUC95lower/AUC)*100)

AUCuppP<-100-((AUC/AUC95upper)*100)

AUC<-(AUC/2000)*100

# Now plot

cols<-colorRampPalette(brewer.pal(9, "Reds"))

thecols<-cols(100)

image(c(0.05,pavals),c(0,abvals),t(AUC),col=thecols[c(round(min(AUC)):round(max(AUC)))],ylab="",xlab="")

mtext("Listening probability",2,2.5,cex=axlabsize)

mtext("Patrol effort",1,2.5,cex=axlabsize)

if(k==1){

if(layoutletter=="B"){

if(th==1){mtext(substitute(paste("Information flow ",italic(T),"=1", sep="")),3,headerheight,cex=headersize)}

if(th==2){mtext(substitute(paste("Information flow ",italic(T),"=2", sep="")),3,headerheight,cex=headersize)}

if(th==1){mtext("(iii)",3,numberheight,cex=numbersize)}

if(th==2){mtext("(iv)",3,numberheight,cex=numbersize)}

}else{

if(th==1){mtext(substitute(paste(" Information flow ",italic(T),"=1", sep="")),3,headerheight,cex=headersize)}

if(th==2){mtext(substitute(paste(" Information flow ",italic(T),"=2", sep="")),3,headerheight,cex=headersize)}

if(th==1){mtext(" (iii)",3,numberheight,cex=numbersize)}

if(th==2){mtext(" (iv)",3,numberheight,cex=numbersize)}

}

}

if (identical(whichlayout,layoutB)==F){

par(mar=c(2,2,2,2))

# Side-plots with the errors

UPPcols<-colorRampPalette(brewer.pal(9, "Blues"))(100)

LOWcols<-colorRampPalette(brewer.pal(9, "Greens"))(100)

image(c(0,abvals),c(0.05,pavals),AUClowP,col=LOWcols[round(AUClowP)],ylab="",xlab="")

image(c(0,abvals),c(0.05,pavals),AUCuppP,col=UPPcols[round(AUCuppP)],ylab="",xlab="")

mtext("L",1,2.5)

mtext("E",2,2.5)

}

legend_image <- as.raster(matrix(rev(thecols), ncol=1))

par(mar=c(3,1,2.5,2))

plot(c(0,2),c(0,1),type='n',axes = F,xlab="",ylab="",main="\n % \nmax. \nAUC")

text(x=1.65, y = seq(0,0.9,l=5), labels = seq(0,100,l=5))

rasterImage(legend_image,0,0,1,0.9,cex=0.8)

} #~~~~~~~~~~~~~~~~~~~~~~~~~~~~~~~~~~~~~~~~~~~~~~~~~~~~~~~~~~~~~~~~~ End of th loop

} #~~~~~~~~~~~~~~~~~~~~~~~~~~~~~~~~~~~~~~~~~~~~~~~~~~~~~~~~~~~~~~~~~~ End of k loop

dev.off()
